# A Systematic Review on the Emerging Fungal Pathogen *Neoscytalidium* Causing Infections Worldwide

**DOI:** 10.1007/s11046-025-00964-4

**Published:** 2025-07-06

**Authors:** Juan José Enriquez-Mendez, Angel Gonzalez

**Affiliations:** 1 Faculty of Medicine, Universidad Cooperativa de Colombia, Medellin, Colombia; 2 Basic and Applied Microbiology Research Group (MICROBA), School of Microbiology, Universidad de Antioquia, Calle 67 No. 53–108; Of: 5–103, Medellin, Colombia

**Keywords:** *Neoscytalidium dimidiatum*, *Neoscytalidium hyalinum*, *Neoscytalidium*, *Scytalidium*, Onychomycosis, Dermatomycosis

## Abstract

Scytalidiosis is a dermatomycosis caused by fungi of the genus *Neoscytalidium*. An increase in the number of cases at the global level has been reported. In the present study, the clinical characteristics of patients diagnosed with scytalidiosis were analyzed through a systematic review of cases reported in the literature. An advanced search was conducted through four databases: MEDLINE/PubMed, SCOPUS, Embase, and SciELO using the terms “*Neoscytalidium*” or “*Scytalidium*”. A total of 155 reports with 5,097 cases were analyzed of which 30.12% were women and 27.31% were men. A total of 37 countries reported cases of scytalidiosis. The USA, Thailand, France, Brazil, Colombia, and the UK had the highest number of cases. The most prevalent species were *N. dimidiatum* (38.96%) and *N. hyalinum* (7.47%). One case of *N. oculus* sp. nov. and seven instances of *N. novaehollandiae* were also reported. Regarding the clinical presentation, 68.30% of patients had onychomycosis, 5.93% had skin infections, and 24.16% presented both types of infections. Other less frequent presentations (1.61%), including keratitis/endophthalmitis, CNS infection, invasive or disseminated infection, sinusitis/rhinosinusitis, mycetoma, endocarditis, and dyskeratosis were also reported. This review shows that the epidemiology of scytalidiosis is changing, other regions that had not been considered endemic are now reporting the highest number of cases. *Neoscytalidium* spp. should be considered an important emerging pathogen and the main non-dermatophyte fungus causing onychomycosis and skin infections after dermatophytes. Likewise, other clinical presentations caused by this fungal pathogen should not be underestimated, especially in patients with some immunocompromise or underlying disease.

## Introduction

*Neoscytalidium* is the causal agent of a dermatomycosis known as scytalidiosis; this dematiaceous fungus belongs to the Botryosphaeriaceae family and is the fungal genus most commonly found in the soil of tropical and subtropical zones [1]. Currently, four species have been recognized within this genus: *N. dimidiatum*, *N. oculus*, *N. novaehollandiae*, and *N. orchidacearum*. The latter two species have been reported to cause infections in plants, while *N. dimidiatum* and *N. oculus* have been associated with infections in humans [2, 3]. The taxonomy of *Neoscytalidium* is very confused; thus, *N. dimidiatum* has been previously known by several names, including *Scytalidium dimidiatum*, *Nattrassia mangiferae*, *Fusicoccum dimidiatum*, and *Hendersonula toluroidea* [4]. Moreover, *N. hyalinum* is considered a closely related species to *N. dimidiatum*, and it has also been reported to cause superficial skin infections and onychomycosis [5]. Although *Neoscytalidium* species are endemic in tropical and subtropical zones, *N. dimidiatum* is most commonly isolated from patients living in Asia, the Indian Ocean, and Central Africa. Meanwhile, *N. hyalinum* is mostly isolated from patients living in the West Indies, South America, and West Africa [6]. Although this mycosis has been rarely described in some European countries, it is generally diagnosed in immigrants from endemic regions.

Infections in humans are usually acquired from the soil and the environment [4, 7]. It is noteworthy, in most cases, the risk factors and clinical presentations of infections caused by *Neoscytalidium* spp. are indistinguishable from those caused by dermatophytes [8–11]. *N. dimidiatum* and *N. hyalinum* are associated mainly with the invasion of the nails, toe webs, and soles of the feet with hyperkeratosis, whereas hands are rarely involved [4]. Disseminated or deep infections occur less frequently but can occur in those patients with underlying diseases and common risk factors for opportunistic fungal infections; therefore, central nervous systems abscesses, endophthalmitis, sinusitis, osteomyelitis, and mycetoma, among other clinical presentations, have been reported [4].

Finally, the treatment of scytalidiosis is quite challenging for several reasons: (1) *Neoscytalidium* is resistant to most antifungals, including azoles (fluconazole, itraconazole, and ketoconazole), allylamines (terbinafine), and griseofulvin; (2) the chronicity of lesions; and (3) the particularities of the affected tissue, for example, the thickness of the nails and skin [4].

In the last decade, an increase in cases of onychomycosis and skin infections, especially those caused by non-dermatophyte fungi, has been observed with a rise in environmental etiological agents such as *Neoscytalidium* spp. This systematic review aims to provide information on infections caused by *Neoscytalidium* spp., the main species involved, demographics, and the major clinical presentations worldwide.

## Methodology

This systematic review followed the Preferred Reporting Items for Systematic Reviews and Meta-Analysis (PRISMA) guidelines. An advanced search was performed with no language or geographic location restrictions through the following databases: Medical Literature Analysis and Retrieval System Online (MEDLINE/PubMed), SCOPUS, Embase, and Scientific Electronic Library Online (SciELO), looking for case reports, observational studies, and clinical trials. Literature was searched from the inception of the databases to September 25, 2024. The used terms were “*Neoscytalidium*” or “*Scytalidium*”.

The information on the selected articles was collected in a data extraction table developed in Microsoft Excel. The extracted information was compared, and disagreements were resolved by consensus. The following information was collected: title, country, year of the reported study, gender, number of people presenting fungal infection, and fungal agent identified as causing infections. Studies that did not detail the presence of onychomycosis; dermatomycosis, or other clinical presentations; studies where onychomycosis or dermatomycosis was caused by other fungal pathogens; as well as systematic reviews, scoping reviews, and narrative reviews were excluded. After reading the titles and analyzing the full text, the most relevant articles for the review that met the inclusion and exclusion criteria were chosen. The review was limited to articles that offered detailed descriptions of agents causing onychomycosis, dermatomycosis, or other unusual clinical presentations. Finally, regarding species identification, in those studies in which the previous taxonomy was used, in the present study the names of the agents were replaced by the current taxonomy. Thus, given the confusing taxonomic classification of *Neoscytalidium* over the past years, and thanks to current molecular taxonomy, and to refine and ensure the search criteria, the terms “*Hendersonula toluroidea”* and “*Nattrassia mangiferae*” were not included in the search criteria. Moreover, *N. mangiferae*, previously widely used to refer to *Scytalidium dimidiatum* isolates, is now considered a distinct species and would be placed in this *Neofusicoccum* group, based on the lack of an arthoconidial anamorph [4]. However, in those studies found with the former term “*H. toluroidea”* used in the search and in which this agent was reported, it was assumed to be under its current name “*Neoscytalidium dimidiatum*.”

## Results

### Study Selection

**Fig. 1:**
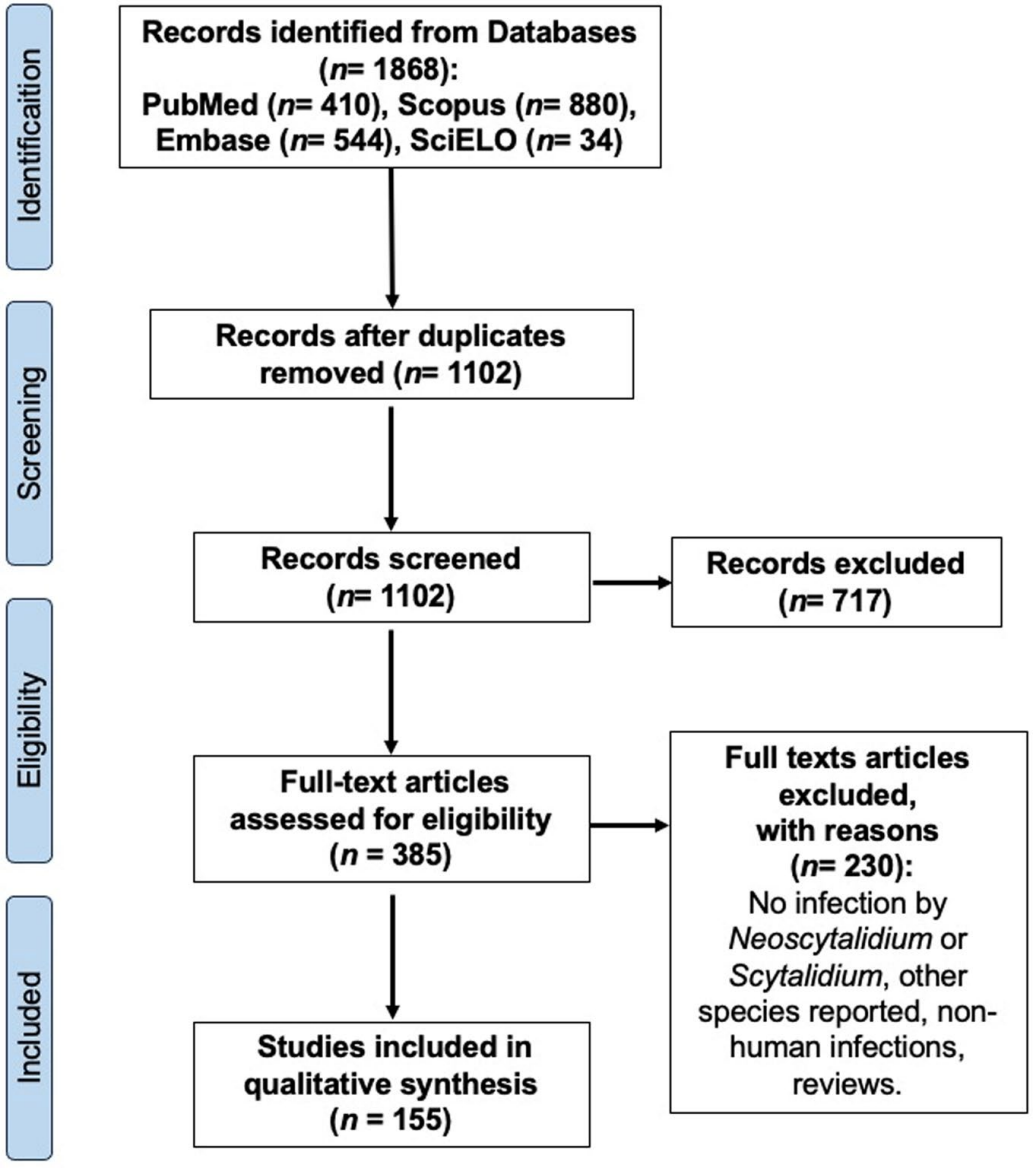
Flowchart with the data selection and analysis methods used in the systematic review

Of the 1868 records identified, 767 were removed due to duplication. After screening the studies by title and abstract, 385 studies remained. Finally, after a full-text assessment, 155 studies were included in the qualitative analysis. The selection process is summarized in Fig. 1.

### Analysis of the Selected Studies

From the 155 studies analyzed, a total of 5097 patients suffering any infection from *Neoscytalidium* spp. were reported at a global level (Table 1). In terms of total cases, 30.12% (n = 1535) of them were women and 27.31% (n = 1392) represented men. Notably, 42.57% (n = 2170) of the patients did not have such information. The mean age of the patients suffering from the mycosis was not able to be analyzed in most of the reports, which is why we decided not to include this information.

**Table 1:** Detailed characteristics of the 155 studies included in the systematic review

Country	Year	Number of patients	Sex F/M	Type or site of infection*	Fungal specie (*n*)	References
Australia	1992	1	ND	Onychomycosis	*N. dimidiatum*	[12]
	1995	6	ND	Onychomycosis, dermatomycosis	*N. dimidiatum*	[13]
	1997	1	0/1	Dermatomycosis	*N. dimidiatum*	[14]
Belgium	2001	4	ND	Onychomycosis	*N. dimidiatum*	[15]
	2012	1	1/0	Onychomycosis, dermatomycosis	*N. dimidiatum*	[16]
Brazil	1999	2	1/1	Onychomycosis	*N. dimidiatum*	[17]
	2005	2	1/1	Onychomycosis	*N. dimidiatum*	[18]
	2007	1	0/1	Onychomycosis	*N. dimidiatum*	[19]
	2008	2	ND	Onychomycosis	*N. dimidiatum* (1)	[20]
					*N. hyalinum* (1)	
	2009	20	ND	Onychomycosis, dermatomycosis	*N. dimidiatum* (18)	[21]
					*N. hyalinum* (2)	
	2010	74	37/37	Onychomycosis, dermatomycosis	*Neoscytalidium* spp.	[22]
	2010	30	ND	Onychomycosis	*Neoscytalidium* spp.	[23]
	2011	30	19/11	Onychomycosis	*Neoscytalidium* spp.	[24]
	2011	1	1/0	Onychomycosis	*N. hyalinum*	[25]
	2012	4	ND	Onychomycosis, dermatomycosis	*N. hyalinum*	[26]
	2013	1	ND	Onychomycosis	*Neoscytalidium* spp.	[27]
	2014	7	ND	Onychomycosis, dermatomycosis	*Neoscytalidium* spp.	[28]
	2017	1	0/1	Onychomycosis	*Neoscytalidium* spp.	[29]
	2017	11	ND	Onychomycosis	*N. dimidiatum* (4)	[30]
					*N. hyalinum* (7)	[31]
	2017	1	0/1	Onychomycosis	*N. dimidiatum*	[32]
	2017	28	ND	Onychomycosis	*N. dimidiatum*	
	2018	30	17/13	Onychomycosis, dermatomycosis	*N. dimidiatum* (24)	[5]
					*N. hyalinum* (6)	
	2018	64	ND	Onychomycosis	*N. dimidiatum*	[33]
	2022	3	3/0	Dermatomycosis	*N. dimidiatum*	[34]
	2023	6	ND	Onychomycosis	*Neoscytalidium* spp.	[35]
Cameroon	2012	10	ND	Onychomycosis	*N. dimidiatum*	[36]
Canada	1989	26	ND	Onychomycosis, dermatomycosis	*N. dimidiatum*	[37]
	2005	1	ND	Onychomycosis	*Neoscytalidium* spp.	[38]
	2008	1	ND	Disseminated infection	*N. dimidiatum*	[39]
	2012	2	ND	Onychomycosis	*Neoscytalidium* spp.	[40]
	2014	2	ND	Onychomycosis	*Neoscytalidium* spp.	[41]
	2016	12	ND	Onychomycosis	*Neoscytalidium* spp.	[42]
Colombia	2004	11	ND	Onychomycosis	*N. dimidiatum*	[43]
	2007	2	ND	Onychomycosis	*N. dimidiatum*	[44]
	2009	7	ND	Onychomycosis, dermatomycosis	*N. dimidiatum* (3)	[45]
					*N. hyalinum* (4)	
	2010	5	ND	Onychomycosis	*N. dimidiatum* (4)	[46]
					*N. hyalinum* (1)	
	2014	178	ND	Onychomycosis, dermatomycosis	*N. dimidiatum*	[47]
	2018	2	ND	Dermatomycosis	*N. dimidiatum*	[48]
	2019	34	ND	Onychomycosis, dermatomycosis	N*. dimidiatum*	[49]
	2019	13	ND	Onychomycosis	*N. dimidiatum* (11)	[50]
					*N. hyalinum* (2)	
	2022	60	31/29	Onychomycosis	*Neoscytalidium* spp.	[51]
Costa Rica	2009	1	1/0	Onychomycosis	*N. dimidiatum*	[52]
Ethiopia	2018	13	ND	Dermatomycosis	*N. dimidiatum*	[53]
	2019	12	ND	Onychomycosis	*N. dimidiatum*	[54]
	2020	11	ND	Onychomycosis	*N. dimidiatum*	[55]
France	1999	3	ND	Onychomycosis, dermatomycosis	*N. dimidiatum*	[56]
	2003	332	ND	Onychomycosis, dermatomycosis	*N. dimidiatum* (154)	[6]
					*N. hyalinum* (178)	
	2004	7	ND	Onychomycosis	*Neoscytalidium* spp.	[57]
	2007	1	1/0	Subcutaneous infection	*N. dimidiatum*	[58]
	2008	32	ND	Onychomycosis, dermatomycosis	*N. dimidiatum* (17)	[59]
					*N. hyalinum* (15)	
	2008	12	ND	Onychomycosis	*N. dimidiatum* (7)	[60]
					*N. hyalinum* (5)	
	2012	21	ND	Onychomycosis, dermatomycosis	*N. dimidiatum* (9)	[61]
					*N. hyalinum* (12)	
	2015	5	1/4	Deep cutaneous infection	*N. dimidiatum* (3)	[62]
					*N. hyalinum* (1)	
					*Neoscytalidium* spp. (1)	
	2022	3	ND	Onychomycosis	*Neoscytalidium* spp.	[63]
French	2011	42	ND	Onychomycosis, dermatomycosis	*N. dimidiatum* (39)	[64]
Guiana					*N. hyalinum* (3)	
Guyana	1984	13	ND	Dermatomycosis	*Neoscytalidium* spp.	[65]
India	1998	1	1/0	Subcutaneous infection	*N. dimidiatum*	[66]
	2007	1	0/1	Dyskeratosis	*N. dimidiatum*	[67]
	2007	2	2/0	Onychomycosis	*N. dimidiatum*	[68]
	2007	1	ND	Onychomycosis	*N. hyalinum*	[69]
	2008	1	0/1	CNS infection	*N. dimidiatum*	[70]
	2010	1	ND	Mycetoma	*N. dimidiatum*	[71]
	2013	1	ND	Onychomycosis	*N. hyalinum*	[72]
	2015	1	ND	Onychomycosis	*Neoscytalidium* spp.	[73]
	2016	7	ND	Onychomycosis	*Neoscytalidium* spp.	[74]
	2016	1	1/0	Endocarditis	*N. dimidiatum*	[75]
	2020	1	0/1	Keratitis	*N. dimidiatum*	[76]
	2022	2	ND	Onychomycosis	*N. hyalinum*	[77]
	2024	1	0/1	Keratitis	*N. dimidiatum*	[78]
Iran	2014	1	0/1	Rhinosinusitis	*N. dimidiatum*	[79]
	2020	1	1/0	Onychomycosis	*N. novaehollandiae*	[80]
	2021	3	3/0	Rhinosinusitis (1), onychomycosis (2)	*N. dimidiatum* (2)	[81]
					*N. novaehollandiae* (1)	
	2021	13	7/6	Respiratory tract	*N. dimidiatum* (8)	[82]
					*N. novaehollandiae* (5)	
	2022	1	0/1	CNS infection	*N. dimidiatum*	[83]
	2022	1	0/1	Onychomycosis	*N. dimidiatum*	[84]
	2022	1	1/0	Rhinosinusitis	*N. dimidiatum*	[85]
Israel	2009	1	0/1	Invasive infection	*N. dimidiatum*	[86]
Italy	1999	2	1/1	Dermatomycosis	*N. hyalinum*	[87]
	2005	2	ND	Dermatomycosis	*N. hyalinum*	[88]
Japan	2022	1	0/1	Dermatomycosis	*N. dimidiatum*	[89]
Korea	2021	1	0/1	CNS infection (Brain abscess)	*N. dimidiatum*	[90]
Kuwait	2009	1	0/1	Dermatomycosis	*N. dimidiatum*	[91]
Lebanon	1984	1	0/1	Subcutaneous infection	*N. hyalinum*	[92]
	2012	1	0/1	Subcutaneous infection	*N. dimidiatum*	[93]
Malaysia	2014	6	ND	Onychomycosis, dermatomycosis	*N. dimidiatum*	[94]
	2017	29	ND	Onychomycosis, dermatomycosis	*N. dimidiatum*	[95]
Martinique	2004	45	ND	Onychomycosis	*N. dimidiatum* (4)	[96]
					*N. hyalinum* (41)	
Mexico	2005	1	0/1	Onychomycosis	*Neoscytalidium* spp.	[97]
	2019	1	0/1	Keratitis	*N. oculus* sp. nov	[3]
Morrocco	2012	1	ND	Onychomycosis	*N. dimidiatum*	[98]
	2018	1	0/1	Endophthalmitis	*N. hyalinum*	[99]
Netherlands	1993	1	0/1	Disseminated infection	*N. dimidiatum*	[100]
Nigeria	1989	31	ND	Onychomycosis, dermatomycosis	*N. dimidiatum* (26)	[101]
	1992	18	ND	Onychomycosis, dermatomycosis	*N. dimidiatum* (13)	[102]
	1997	1	ND	Dermatomycosis	*N. hyalinum*	[103]
Pakistan	1999	1	ND	Onychomycosis	*N. dimidiatum*	[104]
	2003	1	1/0	Mycetoma	*N. dimidiatum*	[105]
	2009	1	0/1	Invasive infection	*N. dimidiatum*	[106]
Saudi Arabia	1993	1	1/0	Endophthalmitis	*N. dimidiatum*	[107]
	2021	1	1/0	CNS infection	*N. dimidiatum*	[108]
	2023	2	1/1	CNS infection	*N. dimidiatum* (1)	[109]
					*N. hyalinum* (1)	
Senegal	2015	1	ND	Onychomycosis	*N. dimidiatum*	[110]
Spain	1984	1	0/1	Onychomycosis, dermatomycosis	*N. dimidiatum*	[111]
	2000	3	2/1	Dermatomycosis	*N. dimidiatum*	[112]
	2004	1	0/1	Endophthalmitis	*N. dimidiatum*	[113]
	2010	9	ND	Onychomycosis, dermatomycosis	*Neoscytalidium* spp.	[114]
	2012	1	ND	Onychomycosis	*N. dimidiatum*	[115]
	2012	1	0/1	Dermatomycosis	*N. dimidiatum*	[116]
	2017	4	2/2	Onychomycosis	*N. dimidiatum* (3)	[7]
					*N. hyalinum* (1)	
Switzerland	2020	18	10/8	Onychomycosis, dermatomycosis	*N. dimidiatum* (14)	[117]
					*N. hyalinum* (4)	
Taiwan	2019	2	1/1	Deep cutaneous infection	*N. dimidiatum*	[118]
Thailand	2004	53	ND	Onychomycosis, dermatomycosis	*N. dimidiatum*	[119]
	2013	1	1/0	Onychomycosis	*N. dimidiatum*	[120]
	2014	41	16/25	Onychomycosis	*N. dimidiatum*	[121]
	2014	1	ND	Onychomycosis	*N. dimidiatum*	[122]
	2015	41	ND	Onychomycosis	*N. dimidiatum*	[11]
	2016	18	ND	Onychomycosis, dermatomycosis	*N. dimidiatum*	[123]
	2016	53	21/32	Onychomycosis	*N. dimidiatum*	[124]
	2016	13	ND	Onychomycosis	*N. dimidiatum*	[125]
	2017	4	4/0	Onychomycosis	*N. dimidiatum*	[126]
	2017	7	ND	Onychomycosis	*N. dimidiatum*	[127]
	2018	13	ND	Onychomycosis	*N. dimidiatum*	[128]
	2018	35	ND	Onychomycosis	*N. dimidiatum*	[129]
	2020	449	ND	Onychomycosis	*N. dimidiatum*	[130]
	2020	32	ND	Onychomycosis, dermatomycosis	*Neoscytalidium* spp.	[131]
	2021	1	ND	Keratitis	*Neoscytalidium* spp.	[132]
	2023	198	ND	Onychomycosis	*Neoscytalidium* spp.	[10]
	2023	184	79/105	Dermatomycosis	*N. dimidiatum*	[133]
Tobago	1984	19	ND	Onychomycosis, dermatomycosis	*N. dimidiatum* (3)	[134]
					*N. hyalinum* (7)	
					*Neoscytalidium* spp. (9)	
			ND			
Tunisia	2000	1	ND	Subcutaneous infection	*N. dimidiatum*	[135]
UK	1977	8		Onychomycosis, dermatomycosis	*N. hyalinum*	[136]
	1979	5	ND	Onychomycosis, dermatomycosis	*N. hyalinum*	[137]
	1984	128	21/107	Onychomycosis, dermatomycosis	*N. dimidiatum* (102)	[138]
	1986	35	ND	Dermatomycosis	*N. dimidiatum* (26)	[139]
	1986	42	6/36	Onychomycosis, dermatomycosis	*N. dimidiatum* (31)	[140]
	1994	12	ND	Dermatomycosis	*N. dimidiatum* (10)	[141]
	1999	1	ND	Onychomycosis	*N. hyalinum*	[142]
	2004	1	0/1	Subcutaneous infection	*N. dimidiatum*	[143]
	2014	1	ND	Sinusitis	*N. dimidiatum*	[144]
	2016	40	ND	Onychomycosis	*N. dimidiatum* (35)	[145]
USA	1994	1	0/1	Subcutaneous infection	*N. dimidiatum*	[146]
	1996	1	0/1	Onychomycosis	*N. dimidiatum*	[147]
	1996	1	1/0	Subcutaneous infection	*N. dimidiatum*	[148]
	2003	1	1/0	Sinusitis	*N. dimidiatum*	[149]
	2006	1	1/0	Keratitis	*Neoscytalidium* spp.	[150]
	2008	1	0/1	Subcutaneous infection	*Neoscytalidium* spp.	[151]
	2011	1	0/1	Pulmonary infection	*Neoscytalidium* spp.	[152]
	2012	1	0/1	Onychomycosis	*N. dimidiatum*	[153]
	2014	1	1/0	Keratitis	*Neoscytalidium* spp.	[154]
	2015	1	0/1	Pulmonary infection	*N. dimidiatum*	[155]
	2016	1	ND	Keratitis	*Neoscytalidium* spp.	[156]
	2017	35	ND	Dermatomycosis (27)	*Neoscytalidium* spp.	[157]
				Invasive infections (3)		
				Respiratory tract (5)		
	2022	1	1/0	Encephalitis	*Neoscytalidium* spp.	[158]
	2024	2171	1232/939	Onychomycosis	*Neoscytalidium* spp.	[159]
Venezuela	2014	2	ND	Dermatomycosis	*N. dimidiatum*	[160]

F, female; M, male; ND, no data; CNS, central nervous system

*Dermatomycosis, in the present study, indicates superficial skin infections

**Fig. 2:**
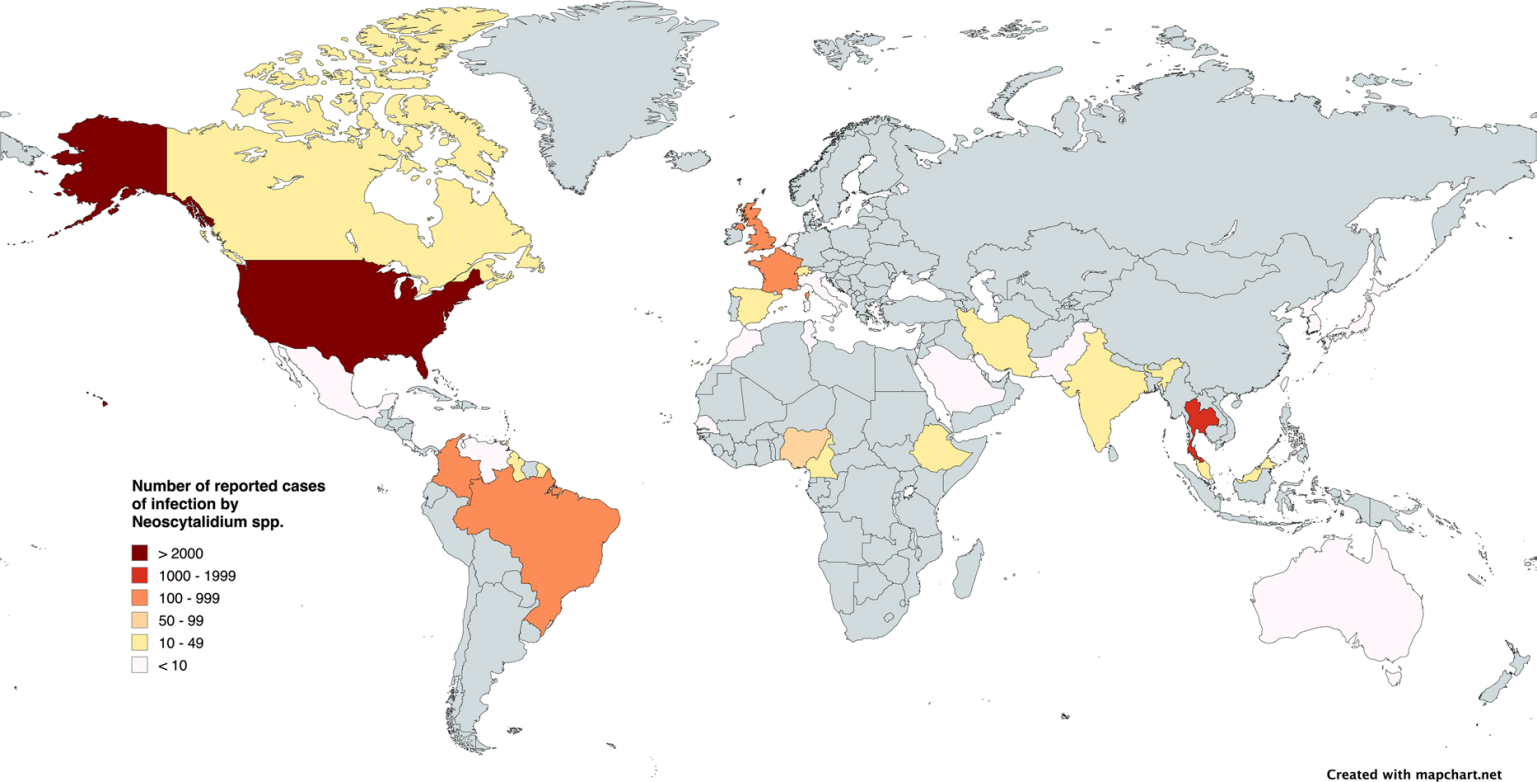
World map estimating the regions most likely to have *Neoscytalidium* infections based on literature reviews. The map was created with mapchart.net

A total of 37 countries, on the five continents, reported cases of scytalidiosis. The six countries with the highest number of reported cases were The United States 43.52% (n = 2218), Thailand 22.44% (n = 1144), France 8.16% (n = 416), Brazil 6.24% (n = 318), Colombia 6.12% (n = 312), and the United Kingdom 5.36% (n = 273) (Fig. 2).

Regarding the clinical presentation, 68.30% of patients (n = 3489) were diagnosed with onychomycosis, 5.93% (n = 303) with dermatomycosis, and 24.16% (n = 1234) presented both onychomycosis and dermatomycosis (Table 1). It is noteworthy that 20 cases (0.39%) presented a pulmonary or respiratory tract infection. Less frequently reported were other clinical presentations that included keratitis/endophthalmitis (n = 10), subcutaneous infection (n = 9), deep cutaneous infection (n = 7), central nervous system infection (encephalitis and abscesses) (n = 7), invasive or disseminated infection (n = 7), sinusitis/rhinosinusitis (n = 7), mycetoma (n = 2), endocarditis (n = 1), and dyskeratosis (n = 1) (Table 1).

**Fig. 3:**
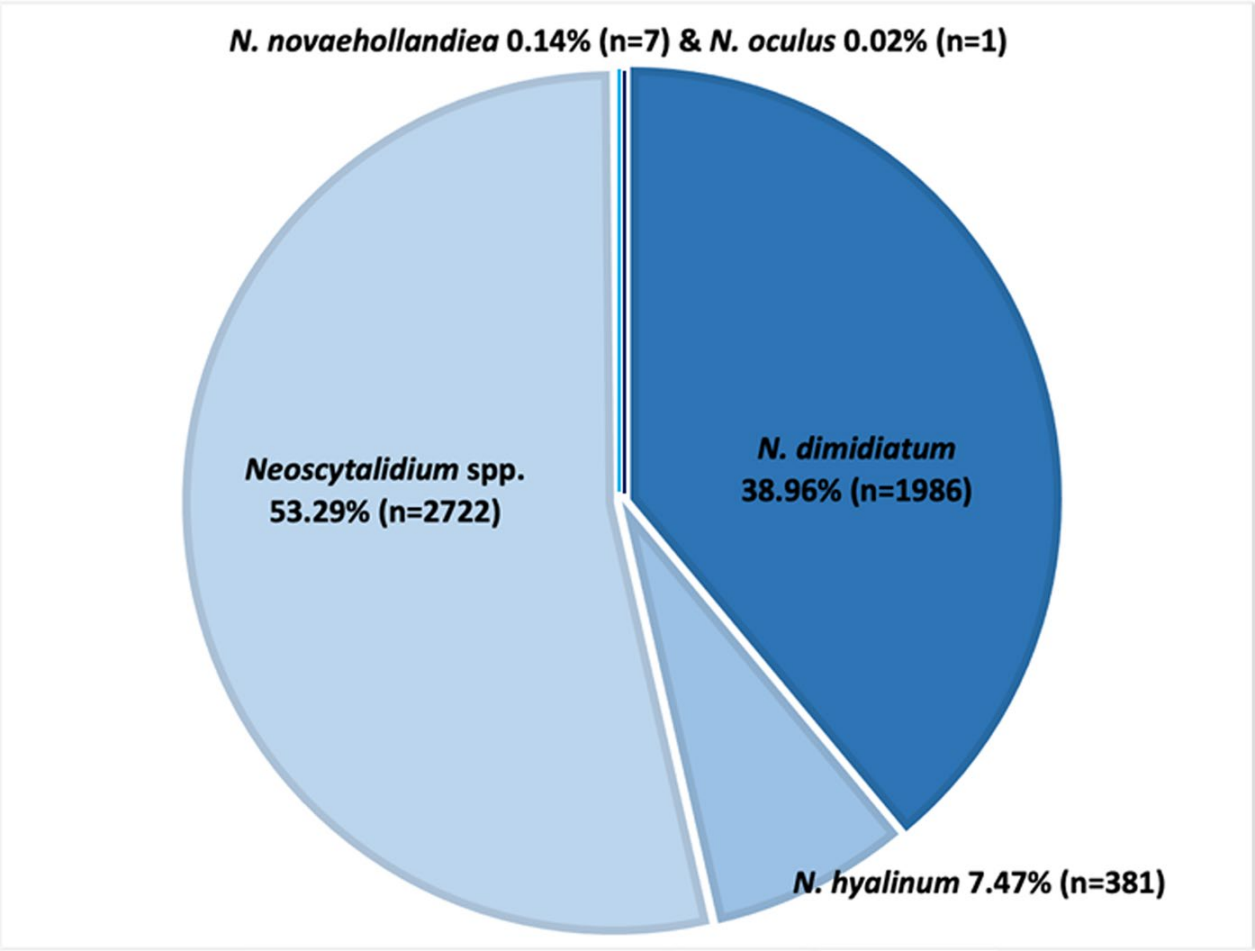
Frequency of species of *Neoscytalidium* reported in this study. A total of 5108 isolates were reported in 155 studies

The causal agents corresponded to *Neoscytalidium* spp. 53.40% (n = 2722), *N. dimidiatum* 38.96% (n = 1986), *N. hyalinum* 7.47% (n = 381), *N. novaehollandiae* 0.14% (7), and *N. oculus* sp. nov. 0.02% (n = 1) (Fig. 3).

## Discussion

*Neoscytalidium* spp. is considered an emerging fungal pathogen, especially causing dermatomycosis, mainly onychomycosis, and skin or cutaneous infections. These filamentous fungi are endemic in tropical and subtropical areas, mostly Africa, South America, the West Indies (Caribbean), India, and Asia, where they commonly represent 40% of dermatomycoses [161]. Notably, in the present study we found that this epidemiology is completely different; thus, several reports indicate that the USA and Thailand, followed by France, Brazil, Colombia, and the UK are the countries with the major number of patients suffering from scytalidiosis (Table 1). These results show an important and changing epidemiology of scytalidiosis, besides indicating the emerging behavior of this fungal pathogen. Accordingly, this change may be attributed to two important factors: (1) the increased migration of people from endemic areas to regions with no previous cases, and (2) climate change, which has been linked to the expanded geographic range of pathogenic fungi, the emergence and reemergence of both new and established pathogens and increased fungal resistance [162].

The increased number of patients reported in the United States representing 43.52% of cases, is largely due to data analyzed by Gupta et al. [159]. In their study, these authors examined 797,560 specimens from 710,541 patients clinically diagnosed with onychomycosis. They found that 2171 patients were infected with *Neoscytalidium* spp., with a higher pathogen prevalence of infection observed in the 45–64-year and ≥ 65-year age groups, although there was no significant difference between sexes. However, the study did not differentiate the implicated species. Similarly, three major studies by Leeyaphan et al. [10, 130, 133], examined a total of 1,588 patients of whom 831 were patients with diagnosed *Neoscytalidium* spp. infections.

France, the country with the third-highest number of patients with scytalidiosis patients reported that most of the patients were in the range of 36 to 65 years old, and a third of the patients were men [6]. It is noteworthy that those patients diagnosed in France were not natives of this country: most came from the West Indies, Sub-Saharan and North Africa, French Guyana, Mauritius, Comoros and Reunion islands, and Asia; while the remaining patients were French residents who had mainly stayed in tropical or subtropical countries [6]. Similarly, patients with scytalidiosis reported in the UK and Spain were immigrants from Africa, the Caribbean, the USA, South America, and Asia [111, 112, 138, 140]. Brazil and Colombia have always been considered endemic areas for *Neoscytalidium* infections; however, they accounted for only 12.36% of the reported cases analyzed in the present study; thus, this suggests that most of the cases could be underreported.

Concerning epidemiology, scytalidiosis is similar in men women; nonetheless, the lack of more information on the occupation of the cases limited a more detailed analysis. However, some reports indicated that occupational activities related to agricultural work were associated with the acquirement of infection [10].

As described in several reports, onychomycosis is the main clinical presentation followed by dermatomycosis, largely with palm and sole lesions. Our findings align with those from the reviewed studies, showing that onychomycosis was the most common clinical presentation, occurring in 68.30% of patients. Skin infections were observed in 5.93% of cases, while 24.16% of patients presented with both nail and skin conditions. Furthermore, other but less frequent clinical presentations were also reported (see Table 1). Among the less frequent clinical presentations, dissemination of the fungus to different organs and systems has been reported; therefore, it could be hypothesized that it only disseminates in patients with other serious underlying diseases that compromise the immune system, similar to what occurs with other opportunistic and environmental fungi.

The fungal species in most of the cases were identified as *N. dimidiatum* (38.96%) or *N. hyalinum* (7.47%); however, in 53.40% of cases, the specific species was not identified. It is important to note that this could be a reflection of the previous difficulty in identifying the species, the confusing taxonomy of *Neoscytalidium*, and the different names that the fungus has received since its first report [4]. Notably, one case was caused by *N. oculus* sp. nov., and seven cases by *N. novaehollandiae*. *N. oculus* sp. nov. was identified as the causal agent of keratitis in a Mexican patient [3]; it is noteworthy that this isolation was confirmed using molecular and phylogenetic analysis that includes the barcode genes encoding the internal transcribed spacer (ITS) 1 and ITS2 of 5.8S ribosomal DNA and the 28S large ribosomal subunit DNA, as well using standard-seq single DNA sequencing [3]. The seven *N. novaehollandiae* isolates were reported as causing onychomycosis only in Iranian patients [80, 81] and were also associated with underlying respiratory diseases including pneumonia, bronchiectasis, cystic fibrosis (CF), and chronic obstructive pulmonary disease (COPD) [82]. Therefore, it can be assumed that *N. novaehollandiae* is endemic only to Iran; however, this species could not be ruled out if it was misidentified in other regions. Interestingly, some reports indicated mixed infection of *Neoscytalidium* spp. concomitantly with dermatophytes including *Trichophyton rubrum* and *T. mentagrophytes* [130, 138].

As it is known, *Neoscytalidium* spp. is resistant to most topical or systemic antifungals used by dermatologists. However, in the study performed by James et al. [95], it was found that clinical isolates of *N. dimidiatum*, in Malaysia, were sensitive to miconazole, clotrimazole, voriconazole, and amphotericin B, in vitro. In another study, Cursi et al. [23] treated 25 patients with onychomycosis caused by *Neoscytalidium* spp. orally with terbinafine plus ciclopirox nail lacquer twice a week for 12 months, with 6 months posttreatment follow-up, and they found a 96% clinical improvement at six months posttreatment. Similarly, Shokoohi et al. [80] treated a patient, with onychomycosis caused by *N. novaehollandiae*, who received oral terbinafine plus ciclopirox nail lacquer twice a week for four months with a complete resolution of the clinical manifestations of onychomycosis. Tonani et al. [5] also reported that amphotericin B, voriconazole, and terbinafine were the most effective antifungals against both *N. dimidiatum* and *N. hyalinum*; additionally, the use of antimicrobial photodynamic treatment was also effective against these two pathogens. Interestingly, Leeyaphan et al. [10] indicated that advanced age, nail thickness, and peripheral vascular disease could be obstacle factors to achieving a complete cure for onychomycosis caused by *Neoscytalidium* spp.

Among the limitations of this study is the lack of important information in most of the reports analyzed, including the age, occupation, treatment, and risk factors, among others.

## Conclusions

This review shows that the epidemiology of scytalidiosis is changing, as other countries or regions that had not been considered endemic, such as the United States, France, Brazil, and Colombia, are now reporting the highest number of cases. Likewise, in European countries, the increase in cases seems not to be due to the presence of the fungus in their environment but to the importation of cases associated with the migration of people, mainly from Africa, Asia, and other endemic tropical countries; however, due to climate change, autochthonous cases should not be ruled out.

*Neoscytalidium* spp. should be considered an important emerging pathogen and the main non-dermatophyte fungus causing onychomycosis and skin infections after dermatophytes. *N. dimidiatum* is the main species causing nail and skin infections; however, the number of *N. hyalinum* has been increasing significantly. Likewise, other clinical presentations caused by this fungal pathogen should not be underestimated, especially in immunocompromised patients or those with underlying diseases.

Interestingly, although *N. novaehollandiae* is a rare species, it has only been reported in Iran associated with respiratory infections, indicating that this country is an endemic area for this species. Given the antifungal resistance of *Neoscytalidium* species, it is crucial to make an accurate diagnosis to guide effective treatment. Therefore, whenever possible, implementing molecular tests is recommended for precise species identification.

## Data Availability

No new data were created or analyzed in this study. Data sharing does not apply to this article.
